# Application of cardiovascular virtual endoscopy: a pilot study on roaming path planning for diagnosis of congenital heart diseases in children

**DOI:** 10.1038/s41598-017-16420-3

**Published:** 2018-01-23

**Authors:** Li-Ping Yao, Ju Mei, Fang-Bao Ding, Li Zhang, Hui-Ming Li, Ming Ding, Xin Yang, Xiao-Ming Li, Kun Sun

**Affiliations:** 1 Department of Cardiothoracic Surgery, Xinhua hospital Affiliated to Shanghai Jiaotong University School of Medicine, Shanghai, 200092 China; 2 Department of Radiology, Xinhua hospital Affiliated to Shanghai Jiaotong University School of Medicine, Shanghai, 200092 China; 3 Department of Pediatric Cardiology, Xinhua hospital Affiliated to Shanghai Jiaotong University School of Medicine, Shanghai, 200092 China; 4 Institute of Image Processing and Pattern Recognition, Department of Automation, Shanghai Jiaotong University, Shanghai, 200240 China; 5 Healthcare Department, Philips Research China, No.10, Lane 888, Tian Lin Road, Shanghai, 200233 China

## Abstract

To investigate roaming paths planning for diagnosis of congenital heart diseases (CHD) using a cardiovascular virtual endoscopy (VE) system. Forty children were enrolled. VE system was applied to support in establishing a diagnosis. Performance in diagnosing CHDs by CT, VE using automatically planned roaming paths (VE-auto, objects were treated as left heart system and right heart system), VE using manually planned paths (VE-manual), and VE using automatically planned path for left heart system and manually planned path for right heart system (VE-combined) were studied and compared. Comparable accuracy of 93%, 93%, 95% and 95% was found by CT, VE-auto, VE-manual and VE-combined. However, in diagnosing tetralogy of Fallot, significantly higher performance was found by VEs, compared with CT. For VE-auto, poor performance with an accuracy of 85% and sensitivity of 22% was revealed in diagnosing muscular ventricular septal defect, compared with VE-manual and VE-combined. Compared with VE-manual, VE-combined illustrated comparable diagnostic accuracy on all CHDs; however, significantly smaller diagnostic time was utilized (*P* < 0.05).Cardiovascular VE system demonstrated considerable clinical value in the diagnosis of CHDs. Left and right heart system should not be modeled as two cavity objects simultaneously. When one of two systems is treated as one object, the other system should be treated as three separate objects when using VE to diagnose CHDs.

## Introduction

Virtual endoscopy (VE) is an application of virtual reality technology in medicine, which is based on computer visualization and modern medical imaging technologies^1^. It usually obtains human anatomic data *via* computer tomography (CT), magnetic resonance imaging (MRI), and ultrasound imaging equipment. Then, with sets of image processing and visualization methods, a three-dimensional (3D) environment similar to that seen through an endoscope is constructed. Therefore, VE allows doctors to explore internal structures in non-invasive ways. To date, several VE systems have been developed including virtual angioscopy, virtual gastrointestinal endoscopy, and virtual bronchoscopy; and these were mainly utilized for pre-surgery planning and education^2–6^. With a totally different field of view, as compared to the conventional CT and MRI, and with advantages in performing measurements, VEs might provide values for the diagnosis and evaluation of diseases. However, few studies have been carried out.

In order to apply VE systems, roaming path planning is vital, directly determines the vision of a doctor, and influences the surgery planning and diagnostic result. Several methods for roaming path generation such as artificial calibration, topological refinement, and distance transformation have been proposed and mainly applied to gastrointestinal applications, where organs with simple cavity structures are involved^7–9^. The proper methods for roaming path planning in cardiovascular applications have rarely been researched.

In this study, a cardiovascular VE system based on cardiac multi-detector computed tomography (MDCT) examinations was developed and applied in the diagnosis of congenital heart diseases (CHD) on 40 children. Three different methods for roaming path planning were designed, and the performance in diagnosing different CHDs was compared with surgical findings as proof.

## Materials and Methods

### Data

This retrospective study was approved by the Institutional Review Board of Xinhua Hospital. Written informed consent was obtained from the parents of all participants. I confirm that all methods were performed in accordance with the relevant guidelines and regulations. From January 2015 to December 2015, a total of 45 children with CHDs were included into this study. All these children received echocardiography and MDCT cardiac examinations before surgery operations. Five cases were excluded due to several artifacts on CT images. Therefore, 40 children were studied. Patient characteristics are illustrated in Table 1. The average age for participants was 7.0 ± 2.9 years (range: 1.3–12.1years). The average CT dose index volume, dose-length of the product, and effective radiation dose was 2.52 mGy (range: 1.76–6.10 mGy), 45 mGy cm (range: 25.6–78.2 mGy·cm), and 0.61 mSv (range: 0.38–1.01 mSv), respectively.

**Table 1:** Characteristics of the study subject with congenital heart disease.

Variable	Value	Diagnosis	No. of patients
General information		ASD/VSD	6
Number (count)	40	ASD/VSD/PDA	6
Age (year, mean ± SD)	7.0 ± 2.9	TOF	3
Male (count, %)	20 (50)	TOF/PDA	2
Radiation dose		TOF/ASD	4
DLP (mGy·cm, mean ± SD)	45 ± 11	TOF/ASD/PDA	2
CTDIvol (mGy, mean ± SD)	2.5 ± 0.7	TOF/VSD/ASD/PDA	7
ED (mSv, mean ± SD)	0.6 ± 0.3	DORV/VSD	2
		DORV/VSD/ASD	5
		C-TGA	1
		D-TGA/VSD/ASD/PS	2

DLP, dose-length product; CTDIvol, CT dose index; ED, effective dose. SD: standard deviation. ASD, atrial septal defect, VSD, ventricular septal defect, PDA, patent ductus arteriosus, TOF, tetralogy of Fallot, DORV, double outlet right ventricle, C-TGA, corrected transposition of great arteries, D-TGA, complete transposition of great arteries, PS, pulmonary stenosis.

Among the 40 cases, each case had more than two types of defects. As confirmed by surgeries, the main diagnosis was as follows: 12 cases had atrial septal defect (ASD) and ventricular septal defect (VSD), 18 cases had tetralogy of Fallot (TOF), seven cases had double outlet right ventricle (DORV), and three cases had transposition of the great artery (TGA). Among these 40 cases, 28 cases had VSD; in which the position of VSD in nine cases was in the muscular interventricular septum, while the rest of the cases were membranous or sub-pulmonary ventricular septal defects.

### MDCT examination and diagnosis

All cardiac CT examinations were performed by a radiologist (Ding M.) who has 10 years of experience in diagnosing CHD using a Philips Brilliance 256-slice CT (Royal Philips, Netherlands). Same setting parameters with collimation of 0.625 mm, rotation time of 270 ms, slice thickness of 0.9 mm, reconstruction interval of 0.45 mm, scan length of 250 mm, resolution of 521 × 512 pixels per slice, and tube voltage of 80 kV were utilized. The contrast agent Iohexol 300 (General Electronics, USA) was injected using a Mallinckrodt binocular high-pressure syringe (injection dose: 2 ml/kg, injection rate: 3.5–5 ml/s) through the median cubital vein. A region of interest (ROI) was selected on the descending aorta for bolus tracking to trigger the scanning. The automatic scanning was triggered in four seconds once the attenuation within that ROI was larger than 80 Hounsfield units (HUs). The temporal window for data acquisition was set at 40–45% of the R-R interval. CT data was recorded in DICOM format for further diagnosis and analysis.

The Cardiac View Software (Royal Philips, Netherlands) was applied on the CT images for post-processing, including curved planar reconstruction, volume rendering and maximum intensity projection (MIP). The diagnosis of CHD by CT was based on the Van Praagh approach^10^, which was performed by a radiologist (Li HM), who has more than 15 years of experience in cardiac applications. The time for each diagnosis was recorded.

### VE system and three patterns for the roaming path

The cardiovascular VE system consisted of three major elements including the automatic segmentation of vessels and heart on 3D image, interactive roaming path planning, and automatic real-time image reconstruction and visualization. This system was developed by Yang X., and was run under the environment of visual studio 2010 on a computer with an Intel i5 processor (2.67 GHz), a DDR3 RAM of 6 GB, and a NVIDIA GTX 465 graphics card.

**Figure 1:**
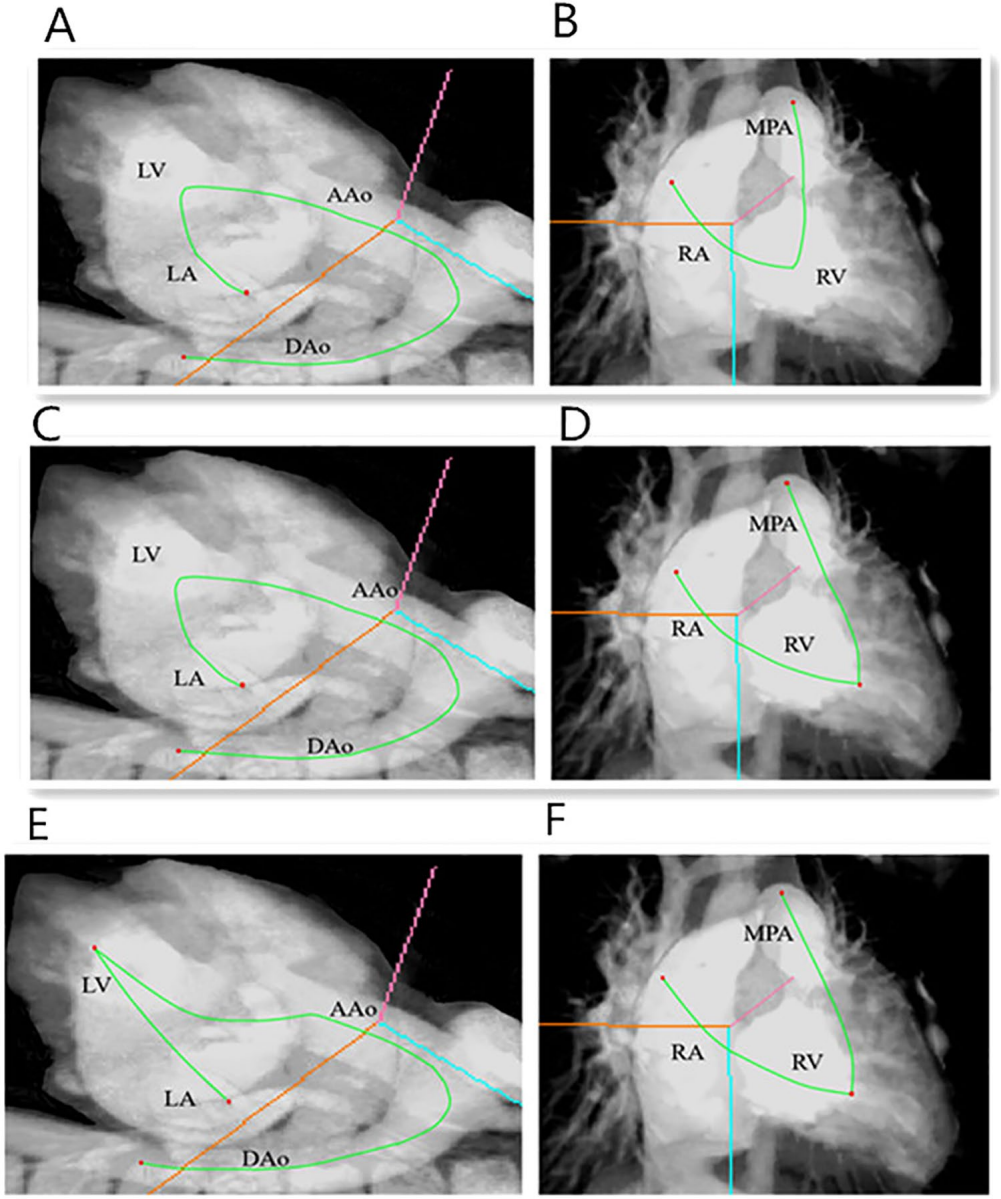
An example of roaming paths plan for left and right heart system in 3D image. Figure **A** and **B** showed the path planned under VE- automatic mode, Figure **A** showed left heart system automatic path and Figure **B** showed right heart system automatic path (green line), two-end seeds (red points) were placed at top of left atrium, midpoint in descending aorta, top of the right atrium and midpoint in the main pulmonary artery. Figure **C** and **D** showed the path planned under VE-combined mode, Figure **C** showed left heart automatic path and Figure **D** showed right heart manual path (green line). Figure **E** and **F** showed the path planned under VE-manual mode, Figure **E** showed left heart manual path and Figure **F** showed right heart manual path (green line), two-end seeds (red points) were placed manually at top point in left atrium, apex point in left ventricle, midpoint in descending aorta, top point in right atrium, apex point in right ventricle and midpoint in the main pulmonary artery, respectively. LA: left atrium; LV: left ventricle; AAo: ascending aorta; DAo: descending aorta; RA: right atrium; RV: right ventricle; MPA: the main pulmonary artery.

For roaming path planning, the two-end seeds method was implemented and integrated into this system^11^, in which the object of interest was modeled as the cavity object; and doctors only needed to mark a start-point and an end-point. With the marked two-end seeds, the distance transformation-based centerline extraction algorithm with iterative centerline smoothing was applied to automatically generate the roaming path from the start-point to the end-point. In this study, the heart was divided into two systems: the left heart system (left atrium, left ventricle and the aortic artery) and right heat system (right atrium, right ventricle and the main pulmonary artery). Three methods for roaming path planning were investigated, as illustrated in Table 2. These include totally automatic path planning (VE-auto), manual path planning (VE-manual), and combined path planning (VE-combined). In VE-auto, the left and right heart system was treated as two separate objects. For the left heart system, the start and end seeds were set at the top point of the left atrium and the point in descending artery, respectively. For the right heart system, the start and end seeds were set at the top point of the right atrium and the point in the main pulmonary artery. Two path segments were generated, as illustrated in Fig. 1A and B. In VE-manual, arteries, atriums and ventricles were modeled as six separate objects, and the start and end seeds were set for each object. Four pathway segments were generated, as illustrated in Fig. 1E and F. In VE-combined, the left heart system was molded as one object, while the rest of the arteries, atriums and ventricles were modeled as three separate objects. The start and end seeds were set for each object. Three pathway segments were generated, as illustrated in Fig. 1C and D.

**Table 2:** Three roaming path patterns designed on VE in CHD diagnosis.

Roaming path patterns	Objects	End seeds	Several Path
VE-auto	left heart system	top point at left atrium - midpoint in descending artery	2
	right heart system	top point at right atrium - midpoint in the main pulmonary artery	
VE-manual	left heart system	top point at left atrium - apex point at left ventricle	4
		apex point at left ventricle - midpoint in descending artery	
	right heart system	top point at right atrium - apex point at right ventricle	
		apex point at right ventricle - midpoint in the main pulmonary artery	
VE-combine	left heart system	top point at left atrium - midpoint in descending artery	3
	right heart system	top point at right atrium - apex point at right ventricle	
		apex point at right ventricle - midpoint in the main pulmonary artery

Two pediatric cardiologists (Yao LP. and Zhang L.) who have more than five years of experience in cardiac applications and blinded to the surgical results were trained to use the VE system and performed the diagnosis using VE-auto, VE-manual and VE-combined, respectively. When inconsistencies occurred, these two cardiologists would reanalyze the image until a consensus was reached. The time for each diagnosis was recorded, and the mean time for every case was used as the diagnostic time.

### Statistics

With surgical results as ground truth, the true positive value (TP), false positive value (FP), truth negative value (TN), false negative value (FN), and accuracy were calculated to evaluate the diagnostic performance by MDCT, VE-auto, VE-manual and VE-combined. Chi-square test was adopted to compare diagnostic accuracy, true positive rate (sensitivity), and true negative rate (specificity) amongst the different methods. Paired two-tailed *t*-test was applied to determine the significance of the difference in diagnostic time through different methods. The difference was considered significant with a *P* value < 0.05. MedCal (10.4.7.0) software was utilized to perform the analysis.

## Results

### Overall diagnostic performance and time on CHDs amongst the different methods

The overall diagnostic performance of CHDs through the different methods is illustrated in Table 3. For MDCT, VE-auto, VE-manual and VE-combined, no significant difference in accuracy (93% *vs*. 93% *vs*. 95% *vs*. 95%, all *P* > 0.05), sensitivity (84% *vs*. 83% *vs*. 89% *vs*. 89%, all *P* > 0.05) and specificity (97% *vs*. 98% *vs*. 98% *vs*. 98%, all *P* > 0.05) were observed.

**Table 3:** Diagnostic performance on CHDs by MDCT and VEs.

Diagnosis	Surgical results	MDCT							VE-auto							VE-manual							VE-combined						
		TP	FP	TN	FN	ACC	SEN	SPE	TP	FP	TN	FN	ACC	SEN	SPE	TP	FP	TN	FN	ACC	SEN	SPE	TP	FP	TN	FN	ACC	SEN	SPE
ASD	32	26	1	7	6	0.83	0.81	0.88	26	3	5	6	0.78	0.81	0.63	26	3	5	6	0.78	0.81	0.63	26	3	5	6	0.78	0.81	0.63
VSD	19	17	1	20	2	0.93	0.89	0.95	17	0	21	2	0.95	0.89	1.00	17	0	21	2	0.95	0.89	1.00	17	0	21	2	0.95	0.89	1.00
m-VSD	9	7	1	30	2	0.93	0.78	0.97	2	0	31	7	0.83	0.22	1.00	7	1	30	2	0.93	0.78	0.97	8	1	30	1	0.95	0.89	0.97
PDA	17	16	0	23	1	0.98	0.94	1.00	16	0	23	1	0.98	0.94	1.00	17	0	23	0	1.00	1.00	1.00	16	0	23	1	0.98	0.94	1.00
PS	2	2	0	38	0	1.00	1.00	1.00	2	0	38	0	1.00	1.00	1.00	2	0	38	0	1.00	1.00	1.00	2	0	38	0	1.00	1.00	1.00
TOF	18	14	2	20	4	0.85	0.78	0.91	17	0	22	1	0.98	0.94	1.00	17	0	22	1	0.98	0.94	1.00	17	0	22	1	0.98	0.94	1.00
DORV	7	5	2	31	2	0.90	0.71	0.94	6	1	32	1	0.95	0.86	0.97	6	1	32	1	0.95	0.86	0.97	6	1	32	1	0.95	0.86	0.97
TGA	3	3	0	37	0	1.00	1.00	1.00	3	0	37	0	1.00	1.00	1.00	3	0	37	0	1.00	1.00	1.00	3	0	37	0	1.00	1.00	1.00
Total	107	90	7	206	17	0.93	0.84	0.97	89	4	209	18	0.93	0.83	0.98	95	5	208	12	0.95	0.89	0.98	95	5	208	12	0.95	0.89	0.98

ASD, Atrial septal defect; VSD, Ventricular septal defect; m-VSD, Muscular ventricular septal defect; PDA, Patent ductus arteriosus; PS, Pulmonary Stenosis; TOF, Tetralogy of Fallot; DORV, Double outlet right ventricle; TGA, Transposition of Great Artery; TP, true positive value; FP, false positive value; TN, true negative value; FN, false negative value; ACC, accuracy; SEN, sensitivity; SPE, specificity.

**Figure 2:**
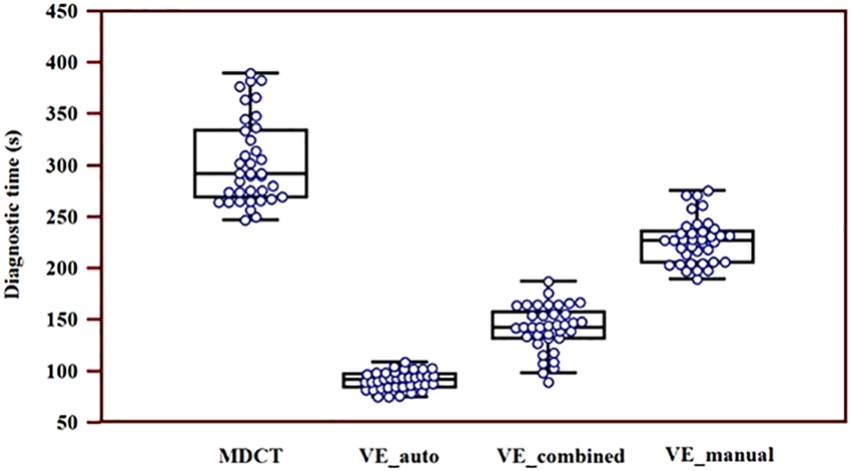
Diagnostic time on CHDs by MDCT and VEs. Significantly shorter diagnostic time was found by VEs as compared to MDCT (153 ± 59 vs. 303 ± 41,* P* < 0.05). Significantly shorter diagnostic time was observed by VE-auto as compared to VE-combined and VE-manual (91 ± 9 vs. 142 ± 22 vs. 226 ± 22s, *P* all < 0.05) and significantly shorter diagnostic time was observed by VE-combined as compared to VE-manual (142 ± 22 vs. 226 ± 22, *P* < 0.05).

The average diagnostic time by MDCT, VE-auto, VE-manual and VE-combined are illustrated in Fig. 2. Significantly shorter diagnostic times were found by VEs as compared to MDCT (153 ± 59 *vs*. 303 ± 41, *P* < 0.05). Furthermore, significantly shorter diagnostic times were observed by VE-auto, as compared to VE-combined (91 ± 9 *vs*. 142 ± 22, *P* < 0.05); and significantly shorter diagnostic times were observed by VE-combined, as compared to VE-manual (142 ± 22 *vs*. 226 ± 22, *P* < 0.05).

### Diagnostic performance on specific CHDs between MDCT and VEs

The diagnostic performance on specific CHDs is illustrated in Table 3. For the diagnosis of TOF, higher sensitivity (94% *vs*. 78%, *P* > 0.05) and accuracy (98% *vs*. 85%, *P* > 0.05) were observed on VEs, as compared to MDCT. For the diagnosis of ASD, lower specificity (63% *vs*. 88%, 5/8 *vs*. 7/8, *P* > 0.05) was observed on VEs, as compared to MDCT. For the diagnosis of other CHDs, similar accuracy, sensitivity and specificity were observed by VEs, as compared to MDCT.

### Diagnostic performance on specific CHDs amongst the three VEs

**Figure 3:**
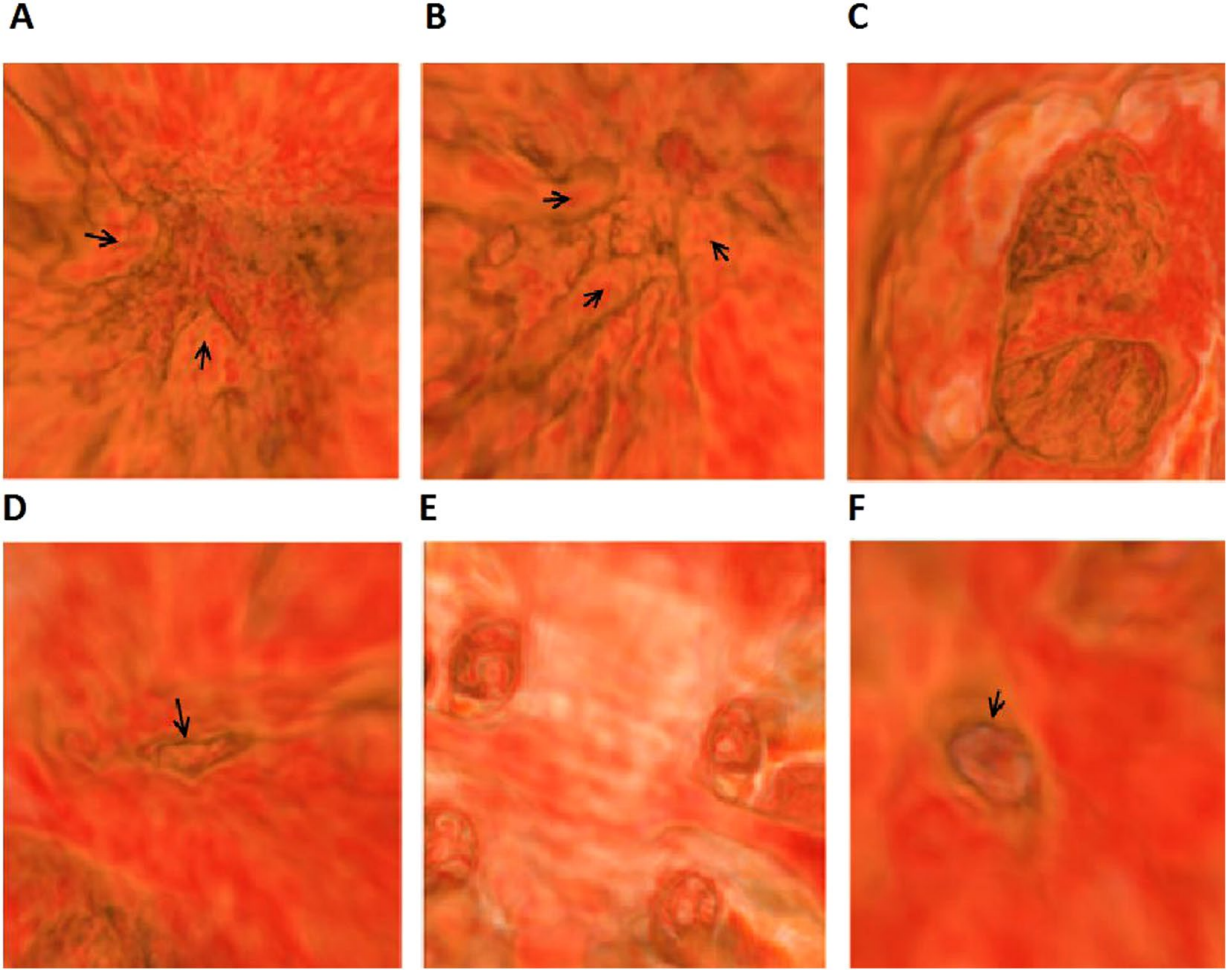
A five-year-old girl diagnosed as TOF with a muscular-VSD under VE. Figure **A** and **B** showed left and right ventricles, there were two groups of papillary muscles (arrow) in the left ventricle and three groups of papillary muscles (arrow) in the right ventricle. Figure **C** showed aortic overriding, figure **D** showed the stenosis mouth (arrow) in right ventricle outflow tract, figure E showed four pulmonary venous openings in left atrium and figure **F** showed a little muscular-VSD (arrow).

For the diagnosis of muscular VSD (Fig. 3F), significantly lower sensitivity (22% *vs*. 78%, 89% and 89%, all *P* < 0.05) and slightly lower accuracy (83% *vs*. 93%, 95% and 95%, all *P* > 0.05) were observed by VE-auto, as compared to MDCT, VE-manual and VE-combined. For the rest of the CHDs, similar accuracy, sensitivity and specificity were observed.

## Discussion

Recently, the clinical values of VE systems for surgical planning and education have been frequently reported^12–15^. However, very few studies have been reported to explore the clinical utilities in the diagnosis and evaluation of certain diseases. In this pilot study, a cardiovascular VE system based on CT images was developed and applied in the diagnosis of different CHDs. Its diagnostic value and methods for the generation of roaming pathways were comprehensively investigated in 40 children with CHDs confirmed by surgery. This study designed three planning methods for roaming path generation: VE-auto (the whole heart was treated as two cavity objects: left heart system and right heart system), VE-manual (artery systems, atriums and ventricles were treated as six separate objects), and VE-combined (left heart system was treated as one cavity object, while the rests were treated as three separate objects).

In our study, the overall diagnostic performance on eight kinds of CHDs was comparable by VEs, as compared to MDCT with similar accuracy (93–95% *vs*. 93%), sensitivity (83–89% *vs*. 84%) and specificity (98% *vs*. 97%). Amongst the CHDs, TOF is the most common one and is the most common cause of pediatric cyanosis^16^. It has been generally regarded to involve four heart anatomical abnormalities presented together, including right ventricular outflow tract obstruction (RVOTO) or pulmonary artery stenosis, ventricular septal defect (VSD), aortic root overriding, and right ventricular hypertrophy. In our study, 18 children were diagnosed as TOF in surgery. The diagnostic performance of TOF by VEs was better than MDCT with higher accuracy (98% *vs*. 85%) and higher sensitivity (94% *vs*. 78%). The diagnosis of TOF, especially the diagnosis of aortic root overriding, provides great benefits due to the advantage of the field of view provided by VE. Using the VE system, a doctor can see directly from the aortic root to the lower ventricles to estimate the degree of overriding, as illustrated in Fig. 3C. For the diagnosis of ASD, a slightly lower specificity was observed by VEs, as compared to MDCT (5/8 *vs*. 7/8). Two cases were missed by VEs, which might be induced by the limitation of the VE system for the final reconstruction and display of the left atrium and right atrium. This could be further improved by reconstruction improvement. Regarding to the diagnostic performance of different methods for roaming pathway generation, similar diagnostic performance was revealed by VE-auto, VE-manual and VE-combined in most of the CHDs, except for the muscular VSD. A significantly lower sensitivity of 22% (2/9) was observed by VE-auto, as compared to VE-manual (sensitivity: 78%, 7/9) and VE-combined (sensitivity: 89%, 8/9). We further reviewed the missed seven cases of muscular VSD by VE-auto, and found that all seven defects were located in the lower region of the ventricular septum. By VE-auto, the generated path couldn’t reach the lower half of the cavity of either the left ventricular or right ventricle, a blind area, located at the lower region of the ventricular septum, was generated in the heart. Therefore, in order to avoid the miss detection of muscular VSDs, the left or right ventricle should not be modeled as one separate object at the same time.

However, significantly lower diagnostic time was achieved through VEs, as compared to MDCT (153 ± 59 *vs*. 303 ± 41, *P* < 0.05). The VE system was established based on MDCT images, and intelligent tools for 3D-visualization and measurements were provided. However, the diagnosis by MDCT continues to depend on 2D CT slices, which is operator-dependent and time consuming.

Our results demonstrate the considerable value of the VE system in the diagnosis of CHDs. For the roaming pathway generation in the diagnosis of CHDs, the left heart system (including the left atrium, left ventricle and aortic artery) or right heart system (including the right atrium, right ventricle and the main pulmonary artery) could be modeled as one cavity object, while the rest should be treated as separate objects.

## Electronic supplementary material

supplementary dataset
